# COVID-19 pandemic and associated factors in pregnant women in urban Bangalore, South India: A qualitative analysis

**DOI:** 10.1192/j.eurpsy.2023.1256

**Published:** 2023-07-19

**Authors:** S. Thomas, K. Srinivasan, T. Thomas, M. Ekstrand

**Affiliations:** 1 Division of Mental Health and Neurosciences, St. John’s Research Institute; 2 Biostatistics, St. John’s Medical College, Bangalore, India; 3 Medicine, University of California, San Francisco, United States

## Abstract

**Introduction:**

The World Health Organization declared COVID-19 as a pandemic in March 2020, and this was followed by a series of preventive measures that included social distancing, travel restrictions and lockdowns in India. Pregnancy is a vulnerable time, with several physical and psychological changes associated with it. The added burden of pandemic could lead to significant stress, and it would be helpful to understand the mitigating factors of stress in this population.

**Objectives:**

The objective of this study was to examine the mitigating factors associated with COVID-19 pandemic among pregnant women.

**Methods:**

The study was conducted in an Urban Primary Health Center (UPHC) in Bangalore that provides maternity care to low-middle income population. Antenatal check-ups are conducted here daily. Pregnant women visiting the clinic for routine antenatal care were approached and informed consent was sought for an interview. 295 women consented to participate in the study. The qualitative interview was conducted in a quiet room. Open ended questions were used to understand the participants’ personal, familial, occupational, and social factors related to COVID-19. The transcripts of the interviews were manually coded for recurring themes by two research assistants. These were examined and similar or identical themes were grouped together. These were further analyzed, and themes were summarized.

**Results:**

The mean (SD) age of the participants was 24.9 (4.2) years, approximately half of the participants were in their first trimester and primiparous. The majority were high school educated and self-employed. 25% of the participants reported mild to moderate depressive symptoms assessed using PHQ-9. The most predominant theme among personal factors was negative emotions that included fear and anxiety. The uncertainty about the transmission and the lack of clarity about the causes during the pregnancy were the reasons for these fears. They reported that they found news and media more stressful. They reported that even though the lockdown restricted their movement, they enjoyed the time they spent with the family, especially their husbands. Most participants reported job loss, theirs and their husbands’ and had to encounter severe economic difficulties. However, the predominant theme was the social support that they received from family, friends, neighbors, and local governing bodies.

**Conclusions:**

Social support was reported to be the most predominant factor that helped the pregnant women to cope with the problems presented during the pandemic. Social support, both instrumental and emotional were important mitigating factors for stress during the pandemic. Strengthening the social support system by support groups and community networking should be a crucial component in government- led initiatives as a factor that may promote resilience in difficult situations like the pandemic.

**Disclosure of Interest:**

None Declared

